# BIOlogical Factors that Limit sustAined Remission in rhEumatoid arthritis (the BIO-FLARE study): protocol for a non-randomised longitudinal cohort study

**DOI:** 10.1186/s41927-021-00194-3

**Published:** 2021-07-19

**Authors:** Fiona Rayner, Amy E. Anderson, Kenneth F. Baker, Christopher D. Buckley, Bernard Dyke, Sally Fenton, Andrew Filer, Carl S. Goodyear, Catharien M. U. Hilkens, Shaun Hiu, Sean Kerrigan, Mariola Kurowska-Stolarska, Fiona Matthews, Iain McInnes, Wan-Fai Ng, Arthur G. Pratt, Jonathan Prichard, Karim Raza, Stefan Siebert, Deborah Stocken, M. Dawn Teare, Stephen Young, John D. Isaacs

**Affiliations:** 1 Translational and Clinical Research Institute, Newcastle University, Framlington Place, Newcastle upon Tyne, NE2 4HH UK; 2 Musculoskeletal Unit, Newcastle upon Tyne Hospitals NHS Foundation Trust, Newcastle upon Tyne, UK; 3 NIHR Birmingham Biomedical Research Centre, University Hospitals Birmingham NHS Foundation Trust and Institute for Inflammation and Ageing, University of Birmingham, Birmingham, UK; 4 Institute of Infection, Immunity and Inflammation, University of Glasgow, Glasgow, UK; 5 Population Health Sciences Institute, Newcastle University, Newcastle upon Tyne, UK; 6 Department of Rheumatology, Sandwell and West Birmingham NHS Trust, Birmingham, UK

**Keywords:** Rheumatoid arthritis, Flare, Remission, Pathogenesis, DMARD withdrawal, DMARD cessation

## Abstract

**Background:**

Our knowledge of immune-mediated inflammatory disease (IMID) aetiology and pathogenesis has improved greatly over recent years, however, very little is known of the factors that trigger disease relapses (flares), converting diseases from inactive to active states. Focussing on rheumatoid arthritis (RA), the challenge that we will address is why IMIDs remit and relapse. Extrapolating from pathogenetic factors involved in disease initiation, new episodes of inflammation could be triggered by recurrent systemic immune dysregulation or locally by factors within the joint, either of which could be endorsed by overarching epigenetic factors or changes in systemic or localised metabolism.

**Methods:**

The BIO-FLARE study is a non-randomised longitudinal cohort study that aims to enrol 150 patients with RA in remission on a stable dose of non-biologic disease-modifying anti-rheumatic drugs (DMARDs), who consent to discontinue treatment. Participants stop their DMARDs at time 0 and are offered an optional ultrasound-guided synovial biopsy. They are studied intensively, with blood sampling and clinical evaluation at weeks 0, 2, 5, 8, 12 and 24. It is anticipated that 50% of participants will have a disease flare, whilst 50% remain in drug-free remission for the study duration (24 weeks). Flaring participants undergo an ultrasound-guided synovial biopsy before reinstatement of previous treatment. Blood samples will be used to investigate immune cell subsets, their activation status and their cytokine profile, autoantibody profiles and epigenetic profiles. Synovial biopsies will be examined to profile cell lineages and subtypes present at flare. Blood, urine and synovium will be examined to determine metabolic profiles. Taking into account all generated data, multivariate statistical techniques will be employed to develop a model to predict impending flare in RA, highlighting therapeutic pathways and informative biomarkers. Despite initial recruitment to time and target, the SARS-CoV-2 pandemic has impacted significantly, and a decision was taken to close recruitment at 118 participants with complete data.

**Discussion:**

This study aims to investigate the pathogenesis of flare in rheumatoid arthritis, which is a significant knowledge gap in our understanding, addressing a major unmet patient need.

**Trial registration:**

The study was retrospectively registered on 27/06/2019 in the ISRCTN registry 16371380.

## Background

**Fig. 1:**
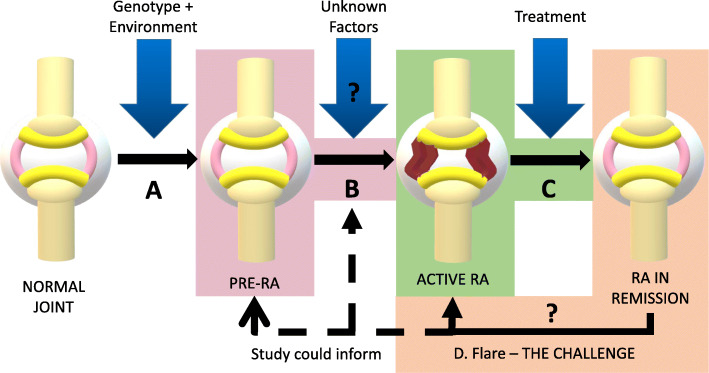
RA Aetiopathogenesis. Environmental factors act upon genetic predisposition to cause breach of tolerance (transition A); Unknown factors act on an individual with pre-RA to trigger active RA (transition B); Treatment induces remission (transition C); Unknown factors trigger flare (transition D). Colours denote what is well investigated (green), the focus of the study (orange) and additional processes that this study could inform (pink)

Rheumatoid arthritis (RA) is a relapsing and remitting autoimmune disease characterised by chronic, polyarticular joint inflammation, disability and premature mortality [1]. Recent epidemiological studies have provided critical aetiological insights that clearly invoke environmental factors, such as cigarette smoking, interacting with an immunogenetic predisposition to trigger breach of tolerance – which, several years later, transitions from asymptomatic ‘pre-RA’ to clinical disease [2, 3]. Once established, the course of RA is usually characterised by episodes of disease remission interspersed with periods of active inflammation, despite the use of synthetic and biological therapies. The pathophysiology of RA remains incompletely understood, but a picture in which disruptions of immune, stromal and epigenetic pathways all contribute to a breakdown in self-tolerance has emerged [4]. However, it is important to note that almost all data underpinning this model are derived from patients with active established disease. In contrast, much less is known about RA in remission and virtually nothing about the processes operating during the transition from remission to flare. This remains a substantially under-investigated area that the BIO-FLARE study aims to address. An understanding of this aspect of RA pathogenesis will prove useful in preventing and treating flares. Beyond this, however, it could also provide insights into the genesis of clinical RA, the critical initiating events wherein the immune system and stroma interact to generate chronic synovial inflammation during the transition from pre-RA to RA (Fig. 1).

The underpinning mechanism(s) of flare have been difficult to study because they occur unpredictably. However, we have recently established a highly relevant human model that generates a ‘synchronized’ population of RA patients in clinical remission, approximately 50% of whom relapse within 6 months, the remainder maintaining remission. The Biomarkers of Remission in Rheumatoid Arthritis (BioRRA) Study sought to identify predictors of drug-free remission in RA. 23/44 (52%) of participants in that study had an RA flare following DMARD cessation [5]. We will study an equivalent cohort of participants in the BIO-FLARE study, to allow us to understand the pathogenesis of flare in RA. Our hypothesis is that, consequent upon epigenetic influences, systemic immune factors integrate with the articular stromal compartment to provoke localised flare, supported and sustained by metabolic changes in the joint. We will use multivariate statistical techniques to develop models to elucidate the longitudinal relationship of potentially pathogenic pathways and identify those that are associated with impending relapse in RA, highlighting tractable therapeutic pathways and informative biomarkers.

Our hypotheses are based on our own published and ongoing studies, in extensive cohorts of early and established RA patients, which we will now apply to patients in remission [6–12]. The key biological question we are addressing is how the known pathways of RA pathogenesis trigger the transition from quiescent to active disease.

## Methods/design

### Study objectives

Our objectives are to carefully study our patient population to address the aforementioned knowledge gap. The primary objective of the study is to measure the immune dysregulation immediately prior to RA flare. In order to interrogate the biology of flare we will focus on:

- Circulating immune cell subsets, their activation status and cytokine profiles
- Autoantibody profiles and other circulating mediators
- Synovial cellular subpopulations
- Metabolic profiles related to immune and/or synovial activation
- Epigenetic profiles

The secondary objectives of the study are:

1. To corroborate baseline biomarkers predictive of sustained DMARD-free remission, as identified by the Biomarkers of Remission in Rheumatoid Arthritis (BioRRA) study
2. To collect biological samples for future discovery research
3. To establish the utility of physical activity monitoring (via accelerometry) to assist in the early detection of flare

### Study design

The BIO-FLARE study is a multi-centre, open-label, prospective, interventional, longitudinal cohort study undertaken at three academic rheumatology centres within the UK (Newcastle, Glasgow and Birmingham). The intervention is complete cessation of non-biological DMARDs (single or combination use of methotrexate, sulfasalazine and/or hydroxychloroquine). All participants who fulfil eligibility criteria will stop their DMARD therapy (without tapering). There is no randomisation involved. Participants will be followed up for 6 months after DMARD cessation or until the point of flare, whichever is sooner.

### Study population

**Fig. 2:**
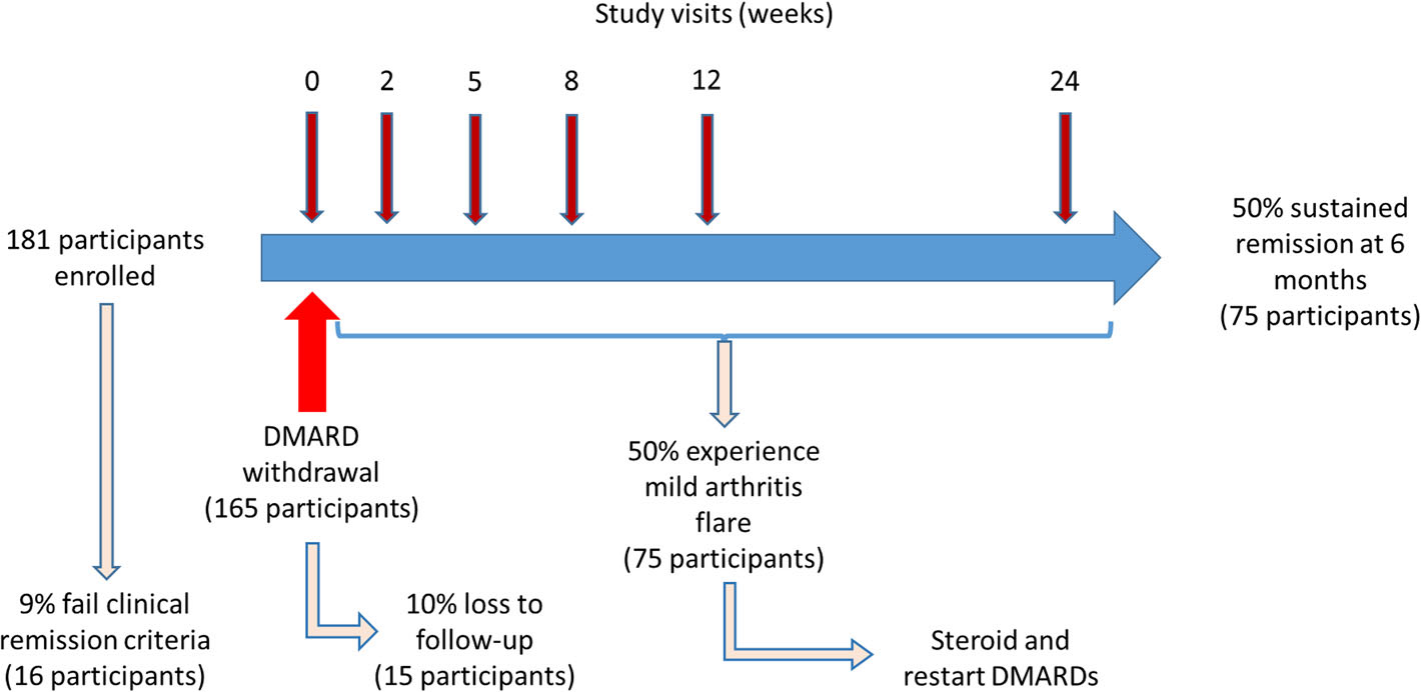
The BIO-FLARE Study overview with estimated patient numbers. Recruitment numbers are extrapolated from data observed in the BioRRA study [5] and other published studies of DMARD withdrawal [13, 14]. Sample collection will occur at baseline, 2, 5, 8, 12 and 24 weeks after DMARD withdrawal, and also at the time of arthritis flare if this occurs

The BIO-FLARE study aims to recruit 150 participants who stop DMARDs and complete the study over a 24-month period. After accounting for withdrawals and subjects who fail to meet the remission criteria, we estimate that we would need to recruit 181 participants to achieve our target (see Fig. 2). Participants may be identified via routine rheumatology outpatient clinics, or through existing databases.

Due to the SARS-CoV-2 pandemic, recruitment to the study was halted from March to August 2020 according to UK Department of Health guidance. After re-opening recruitment, participation remained low, and with increasing infection rates, there was low confidence that the recruitment numbers would improve. After extensive discussion that included trial statisticians, a decision was taken to close recruitment at 118 participants with complete data to enable timely analysis.

### Eligibility criteria

Inclusion criteria

I. Diagnosis of RA according to the 1987 ACR or 2010 ACR/EULAR classification criteria (applied at any time since diagnosis).
II. Current single or combination use of methotrexate, sulfasalazine and/or hydroxychloroquine. No escalations in dose are permitted in the 6 months prior to enrolment, although dose reductions in this time are permitted.
III. Arthritis currently in remission, as judged clinically by referring healthcare professional
IV. Patient and referring clinician willing to consider DMARD withdrawal
V. Age 16 or over at time of symptom onset and 18 or over at time of recruitment

Exclusion criteria

I. Inability to provide informed consent
II. Current participation or follow-up within another ongoing clinical interventional trial
III. Current pregnancy, or pregnancy planned within next 6 months
IV. Major surgery planned within the next 6 months (at discretion of screening clinician)
V. Immunisation within the past 4 weeks
VI. Received oral, parenteral or intra-articular steroids within past 3 months (topical, inhaled and intra-nasal steroids are permitted)
VII. Use of any DMARD other than methotrexate, sulfasalazine or hydroxychloroquine within the past 6 months (or past 12 months for leflunomide)
VIII. Increase in the dose of any DMARD in the 6 months prior to screening
IX. Use of biologic therapy within the past 6 months
X. Prior use of cell-depleting biologic therapies
XI. Haemoglobin < 9 g/L at baseline
XII. Contraindication to synovial biopsy – e.g. bleeding diathesis or prolonged use of anticoagulant therapy (warfarin or other directly acting oral anticoagulants e.g. rivaroxaban)
XIII. Active crystal arthropathy

### Screening visit

Participants attending a screening visit will have the opportunity to discuss the study further with an investigator before signing the consent form. At this point participants are also given the opportunity to consent to additional optional aspects of the study including a baseline synovial biopsy, stool sample collection for microbiome analysis and wearing an accelerometer (GT9X Actigraph) for the duration of the study to enable long-term physical activity monitoring.

Demographic information is collected including RA history, past medical history and medication history. A general physical examination is performed and participants complete questionnaires capturing patient reported outcome measures. Clinical and research blood samples are taken and a urine sample is obtained. A pregnancy test is carried out for women with child-bearing potential. A full list of the schedule of events at each visit is found in Table 1.

**Table 1:** Schedule of events in the BIO-FLARE study

Procedures	Screening Visit	Day 0: Baseline (a) Telephone consultation	Day 0: Baseline (b) Synovial biopsy (OPTIONAL)	Day 14	Day 35	Day 56	Day 84	Day 168	Patient-requested ad-hoc study visits	Visit 2 weeks following ad-hoc study visit	Synovial biopsy assessment visit after flare confirmed
Discuss Study / confirm willingness to continue participation in study	X	X	X	X	X	X	X	X	X	X	X
Informed Consent for study	X										
Collect Demographics and medical history	X										
Record Current medication	X			X	X	X	X	X	X	X	
General Physical examination^a^	X										
Rheumatological Assessment - DAS28-CRP	X			X	X	X	X	X	X	X	
Instruction to discontinue DMARDs (if not opting for synovial Biopsy)		X									
Instruction to discontinue DMARDs (if opting for synovial biopsy)			X								
Patient Reported Outcome Measures / Questionnaires											
HAQ-DI	X							X	X	X	
RAPID-3	X			X	X	X	X	X	X	X	
EuroQol 5D-5L	X			X	X	X	X	X	X	X	
MFI	X							X	X	X	
RA-FQ	X			X	X	X	X	X	X	X	
FLARE-RA	X			X	X	X	X	X	X	X	
Blood tests											
Full Blood Count (FBC)	X			X	X	X	X	X	X	X	
Inflammatory markers (ESR & CRP)	X			X	X	X	X	X	X	X	
Antibodies (RhF & ACPA)	X										
Other clinical bloods (UE, LFT & Clotting)	X								X		
Research blood tests (Serum, EDTA, Tempus and Heparinised samples)	X			X	X	X	X	X	X	X	
Other research tests											
Urine Sample	X			X	X	X	X	X	X	X	
Pregnancy test^b^	X										
Stool Sample (OPTIONAL)	X			X	X	X	X	X	X	X	
Ultrasound assessment for Synovial Biopsy (OPTIONAL AT BASELINE – additional consent required)			[X]								X
Accelerometer provided^c^ (OPTIONAL)	X										
Activity diary provided (OPTIONAL)	X			X	X	X	X		X		

^a^Depending on the circumstances of the consultation, physical examination may be indicated at any study visit to establish whether DAS28-CRP reflects arthritis activity or infection etc. General Physical Examination is only mandatory at Screening

^b^Mandatory at Screening but should be performed at any visit subsequently if routine questioning suggests a participant may be pregnant. Serum or urine tests to be performed subsequently in line with local policy

^c^This may be provided after the study visit once eligibility confirmed, either by post, or at the optional Baseline Synovial Biopsy Visit (if applicable)

Following this visit, the disease activity score in 28 joints with C-reactive protein (DAS28-CRP) is calculated. The cut off for remission was set at DAS28-CRP < 2.4. This is lower than some other remission guidelines given the knowledge that DAS28 values calculated using CRP are commonly lower than those using ESR [15, 16]. If the DAS28-CRP score is < 2.4 and the participant has declined the optional baseline synovial biopsy, then the participant is advised to immediately discontinue their DMARD therapy. Participants who have a DAS28-CRP < 2.4 and have provided additional consent for an optional baseline synovial biopsy will be invited to attend for an additional biopsy visit at their regional Hub site. They will stop their DMARD treatment after the biopsy has been performed. If the DAS28-CRP score is ≥2.4 then the participant is withdrawn from the study at this point, they are referred back to their rheumatology team, and DMARD therapy is continued.

### Follow-up period – day 14, 35, 56, 84, 168 and ad hoc visits

At routine visits, adverse event recording and current medications are noted. Clinical and research blood and urine tests are taken and questionnaires are completed. A joint examination is performed to allow calculation of a DAS28-CRP.

If a participant feels that their arthritis is becoming more active during the follow-up period, then they can arrange an ad hoc visit. These visits follow the same format as the routine visits.

**Fig. 3:**
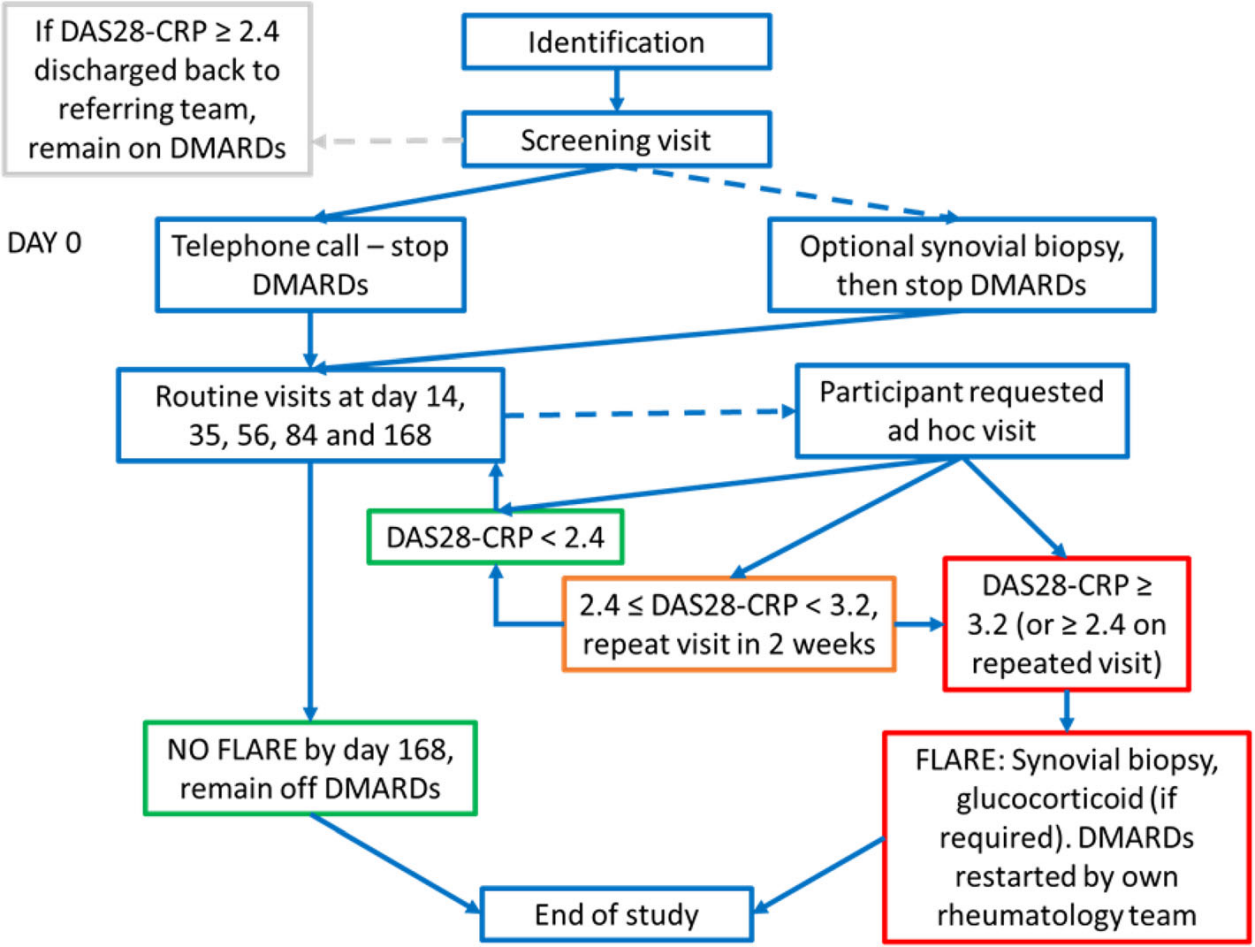
Participant journey through the study. If the DAS28-CRP ≥ 2.4 at the screening visit, the participant is referred back to their own rheumatology team, remaining on DMARDs. All remaining participants stop their DMARDs at day 0 and are followed up with routine visits, or patient requested ad-hoc visits for 24 weeks or until the point of flare

At any point in the study period if the participant is found to have a DAS28-CRP ≥ 3.2 they are defined as having a confirmed flare. If a participant is found to have a DAS28-CRP ≥ 2.4 but < 3.2 they will return for a further ad hoc assessment within 14 days. At the second assessment if the DAS28-CRP ≥ 2.4 they will be defined as a confirmed flare (Fig. 3).

### Confirmed flare

If a participant has a confirmed flare (DAS28-CRP ≥ 3.2, or DAS28-CRP ≥ 2.4 on 2 occasions within 14 days) an ultrasound-guided synovial biopsy will be arranged to take place within 7 days of flare confirmation. Following the synovial biopsy, the participant will receive glucocorticoid therapy if needed, and will be discharged from the study back to their referring team to rapidly recommence DMARDs. A further review (telephone call or face to face visit) will occur 4 weeks after a confirmed flare to ensure the participants’ RA is back under control, that they have had contact with their referring rheumatology team, and that they have resumed DMARD therapy. No study data will be collected at that visit.

### Participant autonomy and safety

The participant has full autonomy throughout this study. The participant information sheet makes it clear that they may withdraw their consent at any time; for example, they may opt not to stop or restart DMARD therapy when it is otherwise indicated to do so in the study protocol. The wishes of the participant will be respected, although the participant will then be discharged from the study and referred back to the clinical (referring) team.

The trial clinician may withdraw a participant from the study at any time if this is considered necessary, and for any reason including:

I. Symptomatic deterioration
II. Participant withdrawal of consent
III. Significant protocol deviation or non-compliance, including failure to attend for more than 2 consecutive visits.
IV. An adverse event, such that discontinuation of DMARDs is no longer appropriate, or renders the participant unable to continue in the trial
V. Termination of the clinical study by the Sponsor
VI. Investigator’s discretion that it is in the best interest of the participant to withdraw

Participants who withdraw from the study will not be replaced automatically as the target sample size has been calculated to allow for withdrawals.

### Clinical discretion

In certain circumstances, it may become clinically apparent that a participant has a DAS28-CRP ≥ 2.4 owing to an alternative clinical diagnosis other than their rheumatoid arthritis. Examples of this may include, but are not limited to:

I. Concurrent infection causing a rise in blood inflammatory markers or patient visual analogue scale leading to a rise in DAS28-CRP above remission thresholds
II. Local trauma event causing joint pain/swelling or a rise in patient visual analogue scale leading to a rise in DAS28-CRP above remission thresholds

In such circumstances, and after agreement with the participant, the research clinician has the discretion not to refer back to the clinical team for restart of DMARD therapy if this is felt to be clinically inappropriate. The participant should continue in the study, but should have an additional appointment review within 14 days. If the participant still has a DAS28-CRP ≥ 2.4 at this additional review, then they should normally be discharged from the study and restart their DMARDs via their referring clinical team.

Similarly, in some circumstances it may become clinically indicated to restart DMARD therapy despite the participant achieving a DAS28-CRP < 2.4 – for example, to control extra-articular manifestations of RA not measured in the remission criteria, or flaring in non-DAS28 joints, e.g. the ankles, feet. In these circumstances, the research clinician has the discretion to refer back to the clinical team for restart of DMARD therapy if this is felt to be clinically indicated at that time. The participant would then also be discharged from the study.

Furthermore, the clinician has the discretion to arrange additional investigations on clinical grounds as required in response to adverse events.

### Adverse events

Adverse events will be recorded at each study visit; the investigator will determine the severity as mild, moderate or severe, and ascertain any causal relationship with the study intervention. Any serious adverse events will be reported immediately to the chief investigator who will inform the Sponsor and the Research Ethics Committee (REC). A serious adverse event is any adverse event which:

- Results in death
- Is life-threatening (i.e. an event in which the participant was at risk of death at the time of the event; it does not refer to an event which hypothetically might have caused death if it were more severe)
- Requires hospitalisation, or prolongation of existing hospitalisation
- Results in persistent or significant disability or incapacity
- Is a congenital anomaly or birth defect

Following cessation of DMARD therapy, we expect that approximately half of the participants will experience a confirmed flare of their arthritis requiring resumption of DMARD therapy and potentially temporary glucocorticoid treatment, without the need for hospitalisation. We also expect that participants who consent to synovial biopsy may experience temporary symptoms (24–48 h) of mild joint pain, swelling or bruising at the biopsy site, but that this is a safe and well tolerated procedure [17]. Joint pain, joint swelling and joint stiffness will not be recorded as an adverse event throughout the BIO-FLARE study.

### Pregnancy

Pregnancy is associated with a diverse range of physiological changes, and is often associated with a reduction in rheumatoid arthritis activity. Pregnancy can therefore be expected to greatly influence the underlying pathogenic processes in RA and any associated biomarkers, and thus pregnancy is an exclusion criterion for this study.

Where pregnancy in a female participant becomes apparent after the enrolment of the participant to the study, this must be recorded as an adverse event. The participant’s referring rheumatologist and GP must be informed, and then the participant must be discharged from the study, remaining off DMARD therapy. The progress of the pregnancy should be followed to term, with no additional follow-up required if the neonate is healthy at birth.

Where the female partner of a male study participant becomes pregnant during study participation, this should also be recorded as an adverse event. The participant may continue within the study. The progress of the pregnancy should be followed to term, with no additional follow-up required if the neonate is healthy at birth.

### Trial governance

The trial management group (TMG) functions to provide operational oversight of the entire BIO-FLARE project including patient recruitment, clinical procedures, laboratory procedures and finance. Any changes to the study Protocol, Participant Information Sheet, Consent Form or Standard Operating Procedures must be reviewed and authorised by the TMG prior to implementation.

As this is a non-CTIMP study, a Combined Trial Oversight Committee (CTOC) will subsume the roles of a Trial Steering Committee (TSC) and Data Monitoring and Ethics Committee (DMC). The CTOC provides independent oversight of the BIO-FLARE project, particularly with regard to patient recruitment and safety. Independence is to be maintained by the use of open and closed sessions for the CTOC and by limiting the voting rights of the non-independent representatives.

### Statistical considerations

The aims of the statistical analyses are to estimate and understand the longitudinal stochastic relationship between the risk of flare and each of the markers in the relevant biological pathways. The overall proportion (confidence interval) of participants who flare by 24 weeks will be estimated using the actual time of flare based on Kaplan-Meier estimates. Graphical representation and descriptive statistics of the longitudinal marker measurements will indicate trends in markers over time, to identify plausible differences in trends in participants who flare and those who do not.

The association between markers and risk of flare will be investigated in a series of descriptive univariate models, based on analysis of each covariate in Cox proportional hazards models, or parametric alternatives, with time varying covariates to account for the longitudinal nature of marker data, through attributing variability at the participant level, time level and marker level. Individual covariates may not be linear in their relationship with outcome so the ‘best’ fitting non-linear relationship will be explored using Fractional Polynomial transformation [18]. The magnitude of the relationship of each marker with risk of flare will be described using estimated beta coefficients (with confidence intervals) accounting for variability at the time level and marker level.

### Data handling

Data, including the number of patients screened, approached and interested in taking part, will be collected via a log completed by staff conducting screening. Trial data for individual participants will be collected by each Principal Investigator (PI) or their delegated person and recorded in the electronic case report form (eCRF) for the trial. Participant identification within the eCRF will be through a unique trial identifier number. A record linking the participant’s name to the unique trial identifier number will be held only in a locked room at the trial site, and is the responsibility of the PI. As such, participants cannot be identified from eCRFs. The Chief Investigator (CI) or delegated person will monitor completeness and quality of data recording in eCRFs and will correspond regularly with site PIs (or their delegated team member) with the aim of capturing any missing data where possible, and ensuring continuous high quality of data. No participant identifiable data will leave the study site. The quality and retention of study data will be the responsibility of the CI. All study data will be retained in accordance with the latest directive on Good Clinical Practice (GCP) and local policy.

All trial data will be stored securely in accordance with GCP, and the Sponsor and Newcastle Clinical Trials Unit (CTU) Standard Operating Procedures (SOPs). Any personal identifiable information will be stored at the study site for 10 years before secure disposal. Data will be handled, computerised and stored in accordance with the Data Protection Act 2018.

## Discussion

The outlook for patients diagnosed with RA now is vastly different compared with 30 years ago. With rapid access to diagnosis and treatment, a large proportion of patients with a new RA diagnosis can hope to achieve remission or low disease activity [19]. The widespread use of methotrexate and other disease modifying drugs has changed the face of this chronic disease, which traditionally resulted in chronic pain and disability. More recently, the advent of biologic drugs has transformed the treatment of those with moderate to severe disease, allowing many patients to continue their normal lives [20]. However, a large portion of patients who achieve remission, subsequently flare. The unpredictable nature of flares can cause disruption in home and working lives, and there is a well-documented association between rheumatoid arthritis and levels of anxiety and depression [21]. This unpredictability also makes the scientific investigation of flares very difficult. There is a gap in our knowledge of what triggers flares, and what pathophysiological changes happen during the flare process. RA is just one example of an immune-mediated relapsing remitting disease. If more was understood about the process of flare in RA, it could help to inform disease flares in other IMIDs such as multiple sclerosis, inflammatory bowel disease and psoriasis.

Our previous study investigating the biomarkers of remission in rheumatoid arthritis (BioRRA study) studied a similar cohort of patients, half of whom flared over a 6-month period [5]. This finding is comparable with other similar studies into remission and flare [13, 14, 22]. There is always an ethical consideration in a study of this nature, given that patients who are previously well, with their disease in remission, are asked to stop their medication. However, experience and our prior work tell us that a significant number of patients are keen to do this [23]. When consulting with our Newcastle Patient and Public Involvement (PPI) group in the planning stages, there was great appetite for such a study. Patients might be experiencing side effects from their medication, or the undesirable need for ongoing blood tests. Some patients simply do not wish to take a medication regularly, particularly if their disease has been quiescent for years, as they may feel the medication is not needed. As a speciality, rheumatologists are becoming more familiar with withdrawing medications when patients are in remission, and indeed, DMARD minimisation (though not necessarily complete cessation) in the setting of RA remission is now supported by current RA management guidelines issued by the American College of Rheumatology (ACR) [24], and the European Alliance of Associations for Rheumatology (EULAR) [25].

The design of the BIO-FLARE study allows a participant to be closely monitored in the period immediately prior to clinical onset of flare. Clinical determinants are collected and tracked over time, such as CRP, joint counts and RAPID-3 scores, along with biological outcomes. At each participant visit, blood is taken for research, processed following SOPs and stored, with the sample location information recorded in a laboratory inventory management system (LIMS). The downstream analysis of this blood will allow multiple questions to be explored. Epigenetic profiling of PBMCs and CD14+ monocytes will be conducted allowing comparison to those who stay in remission. Whole blood and PBMCs will be analysed by flow cytometry to provide deep immune-phenotyping of the samples. Serum and urine samples will be used for metabolic profiling, and autoantibody profile and cytokine levels will also be investigated using serum samples. Synovial tissue collected at the point of flare will be analysed and, in certain cases, can be paired with samples from the same participant whilst in remission, prior to DMARD cessation.

The impact of the SARS-CoV-2 pandemic has had far reaching consequences for all aspects of medical care, including non-COVID research. After extensive discussions amongst the Trial Management Group, including statisticians, the decision was made to close recruitment at 118 participants to enable timely data analysis. Strategies to lessen the impact caused by the reduced sample size on power will be considered including, for example, restricting the number of biomarkers to be considered in a multivariable risk model.

In summary, the novel nature of this study will allow deep interrogation of the mechanisms occurring in the days and weeks leading up to the point of RA flare. Our aim is to further the understanding of the pathophysiology of flare, develop strategies for intervention, and also to prevent or predict flare. Furthermore, these same mechanisms may also be at play during the onset of RA, and thus may also serve to further our understanding of the immunopathology of early stages of the disease.

## Data Availability

Not applicable

## Abbreviations

ACPA: Anti-citrullinated peptide antibody
ACR: American College of Rheumatology
CI: Chief Investigator
CRP: C-reactive protein
CTOC: Combined Trial Oversight Committee
CTU: Clinical Trials Unit
DAS28-CRP: Disease Activity Score using 28 joint count and CRP
DMARD: Disease modifying anti-rheumatic drug
eCRF: Electronic case report form
ESR: Erythrocyte sedimentation rate
EULAR: European Alliance of Associations for Rheumatology
FBC: Full blood count
GCP: Good clinical practice
HAQ-DI: Health assessment questionnaire-disability index
HRA: Health research authority
IMID: Immune mediated inflammatory disease
LFT: Liver function tests
LIMS: Laboratory inventory management system
MFI: Multidimensional fatigue inventory
PBMC: Peripheral blood mononuclear cell
PI: Principal investigator
PPI: Patient and public involvement
RA: Rheumatoid arthritis
RA-FQ: Rheumatoid arthritis flare questionnaire
RAPID-3: Routine assessment of patient index data-3
REC: Research Ethics Committee
RhF: Rheumatoid factor
SOP: Standard operating procedure
TMG: Trial Management Group
UE: Urea, electrolyte and renal profile
